# *PTPN2*, A Key Predictor of Prognosis for Pancreatic Adenocarcinoma, Significantly Regulates Cell Cycles, Apoptosis, and Metastasis

**DOI:** 10.3389/fimmu.2022.805311

**Published:** 2022-01-27

**Authors:** Wenbin Kuang, Xiao Wang, Jiayu Ding, Jiaxing Li, Minghui Ji, Weijiao Chen, Liping Wang, Peng Yang

**Affiliations:** ^1^ State Key Laboratory of Natural Medicines and Jiangsu Key Laboratory of Drug Design and Optimization, China Pharmaceutical University, Nanjing, China; ^2^ Department of Medicinal Chemistry, School of Pharmacy, China Pharmaceutical University, Nanjing, China

**Keywords:** pancreatic cancer, PTPN family genes, PTPN2, prognosis, JAK-STAT

## Abstract

**Objective:**

This study conducted a comprehensive analysis of the members of the PTPN family and emphasized the key role of *PTPN2* as a potential therapeutic target and diagnostic biomarker in improving the survival rate of PAAD.

**Method:**

Oncomine was used to analyze the pan-cancer expression of the PTPN gene family. The Cancer Genome Atlas (TCGA) data as well as Genotype-Tissue Expression (GTEx) data were downloaded to analyze the expression and prognosis of PTPNs. The diagnosis of PTPNs was evaluated by the experimental ROC curve. The protein-protein interaction (PPI) network was constructed by combining STRING and Cytoscape. The genes of 50 proteins most closely related to PTPN2 were screened and analyzed by GO and KEGG enrichment. The differentially expressed genes of *PTPN2* were found by RNA sequencing, and GSEA enrichment analysis was carried out to find the downstream pathways and targets, which were verified by online tools and experiments. Finally, the relationship between *PTPN2* and immune cell infiltration in PAAD, and the relationship with immune score and immune checkpoint were studied.

**Result:**

The expression patterns and the prognostic value of multiple PTPNs in PAAD have been reported through bioinformatic analyzes. Among these members, *PTPN2* is the most important prognostic signature that regulates the progression of PAAD by activating JAK-STAT signaling pathway. Comparison of two PAAD cell lines with normal pancreatic epithelial cell lines revealed that *PTPN2* expression was up-regulated as a key regulator of PAAD, which was associated with poor prognosis. Knockdown of *PTPN2* caused a profound decrease in PAAD cell growth, migration, invasion, and induced PAAD cell cycle and apoptosis. In addition, we conducted a series of enrichment analyses to investigate the *PTPN2*-binding proteins and the *PTPN2* expression-correlated genes. We suggest that *STAT1* and *EGFR* are the key factors to regulate *PTPN2*, which are involved in the progression of PAAD. Meanwhile, the silencing of *PTPN2* induced the repression of *STAT1* and *EGFR* expression.

**Conclusion:**

These findings provide a comprehensive analysis of the PTPN family members, and for PAAD, they also demonstrate that *PTPN2* is a diagnostic biomarker and a therapeutic target.

## Introduction

pancreatic adenocarcinoma (PAAD) is notorious for ranking the highest mortality rates of any solid tumor (1, 2). PAAD is difficult to detect early, it does not cause symptoms immediately. Compared to other solid tumors, PAAD has the characteristics of high malignant rate, low resection rate, high recurrence rate, and poor prognosis. In 2020, the relative 5-year survival rate is less than 9% (3, 4). Because PAAD is usually discovered late and spreads quickly, treatment is difficult (5, 6). Diagnosing pancreatic cancer is challenging, and most cases occur in advanced stages or locally advanced or metastatic disease. There are many reasons for this, including non-specific symptoms related to the disease, as well as proximity to major blood vessels that can easily be infected by tumors. These factors mean that 80-85% of tumors cannot be removed once they appear. Currently, surgical resection is the only possible treatment for pancreatic cancer, although the recurrence rate is high, and the long-term survival rate is inevitably low. Recently years, researchers are studying new therapies including immunotherapy and targeted therapy, but many drugs have not produced satisfactory results (7–9). Therefore, it is urgent to develop new and reliable biological treatment targets for PAAD.

Phosphorylation is a post-translational modification process which is universal and reversible. It plays critical roles in plenty of cellular regulations, such as cell growth, metabolism, and communication, and changes the activity of downstream targets in cell signaling pathways (10, 11). The abnormal regulation of phosphorylation is related to the occurrence of many human diseases, including malignant tumor, neurodegeneration, inflammatory disease, and diabetes (12–14). Protein tyrosine phosphatases (PTPs) take part in the dephosphorylation of tyrosine residues, acting as protein tyrosine kinases (PTKs) to maintain the normal signaling transduction (15, 16). PTPNs are parts of the biggest family class I PTPs, which have been found to act on many important biological processes, including tumor metastasis, cell survival, apoptosis, migration, immune response (17–19). Currently published studies have shown that PTPN family genes participate in the occurrence and development of numerous cancers (20–22). For example, *PTP1B*, encoded by the oncogene *PTPN1*, plays a significant role in the regulation of the AMPK pathway and reduces the proliferation of PAAD cells (23). Recently in human colorectal cancer (CRC) tissue, the higher expression of *PTPN2* with the reduction of anti-tumor immunity (24). In addition, one study suggested that the loss of *PTPN2* could improve the therapeutic efficacy of CAR-T cells in malignant tumors (25). The growth of liver cancer can be promoted by overexpression of *PTPN11* (26). *PTPN12* plays an important regulatory role in tumor deterioration and triple-negative breast cancer (TNBC) transformation (27, 28). Increasing evidences have shown that dysregulation of *PTPN22* offers the potential to improve cancer immunotherapy not only by regulating IFNAR and TCR signaling pathway (29), but also includes the activation of 3 inflammasomes through the regulation of autophagy and the pyrin domain of the NLR family (30). Generally, these evidences make PTPN genes promising prognostic and therapeutic targets for cancer treatment. However, the unique function of PTPN family genes in PAAD has not been fully elucidated so far.

Herein, on the basis of bioinformatics analysis and validation assays, we first did an analysis on prognostic value and expression patterns of various PTPN genes in PAAD. Especially, we found the upregulation of *PTPN2* was associated with adverse prognosis and emerged as a critical regulator of PAAD. Furthermore, knockdown of *PTPN2* decreased the growth, migration, invasion, and induced apoptosis of PAAD cells significantly. We also analyzed the targeting PTPN2-binding proteins and the *PTPN2* expression-correlated genes and found that *STAT1* and *EGFR* are key factors for *PTPN2* to regulate the deterioration of PAAD. Taken together, these findings provided a comprehensive analysis of the PTPN family members and shed light on the critical role of *PTPN2* as a potential therapeutic target and diagnostic biomarker for improving PAAD survival.

## Results

### PTPNs Transcription Level in Pancreatic Adenocarcinoma Patients

**Figure 1:**
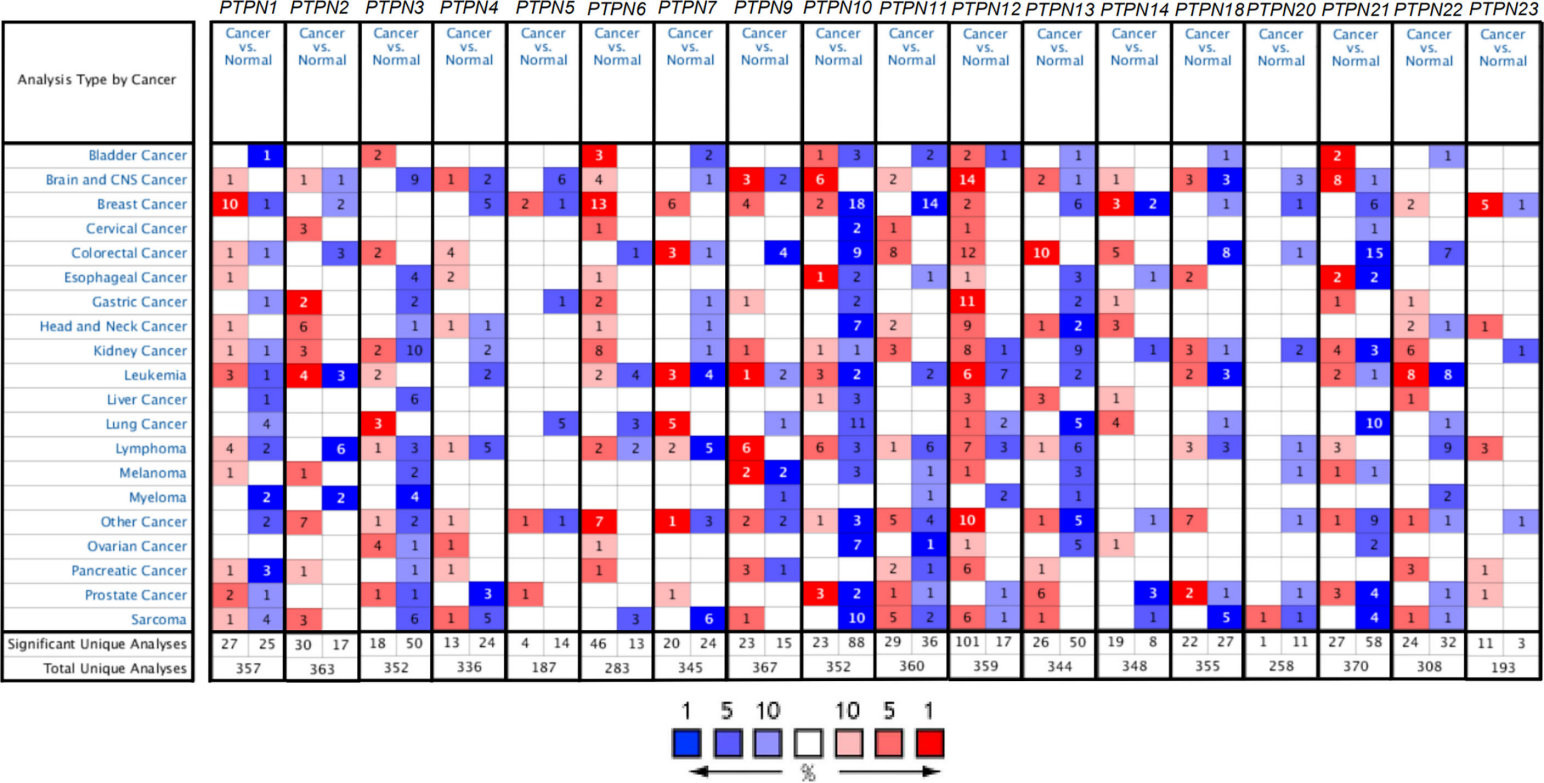
The expression of PTPNs in various tumor tissues and normal tissues in the Oncomine database. Red and blue represent datasets of cancer and normal tissues, respectively. The darker color illustrates that the gene was ranked at approximately the top of the expression quantity in this tumor. The rank for a gene is the median rank for that gene across each of the analyses. And the number in the square represents the number of datasets that met our screening requirements (P < 0.05).

In mammalian cells, there have identified eighteen members of the PTPN family. To study the different expressions of PTPNs in various tumor and normal tissues, we first compared all PTPN family members’ transcription levels using the Oncomine database (**Figure 1**). For PAAD patients, the mRNA expression level of PTPNs is significantly upregulated. In the Grutzmann dataset, compared to normal tissue, *PTPN1* was overexpressed in pancreatic ductal adenocarcinoma epithelia (fold change = 1.542) (**Table 1**). Ishikawa dataset showed that in pancreatic ductal adenocarcinoma *PTPN2* was increased with a fold change of 1.895 when compared with normal samples (**Table 1**). *PTPN4* expressed higher in pancreatic ductal adenocarcinoma with a fold change of 1.625 versus normal samples. For *PTPN6*, its expression level is higher in PAAD with a fold change of 1.754 versus normal samples. Especially for *PTPN9*, its expression level is higher in PAAD with a fold change of 2.229 in Iacobuzio-Donahue dataset, the fold change of expression level in pancreatic ductal adenocarcinoma is 1.664 in Grutzmann dataset, and the fold change in pancreatitis is 2.697 in Logsdon dataset versus normal samples. In addition, Ishikawa dataset reported that in pancreatic ductal adenocarcinoma *PTPN11* was overexpressed (fold change = 1.602), and the analysis of Grutzmann dataset revealed that expression of *PTPN11* in pancreatic ductal adenocarcinoma was also increased with a fold change of 2.122 versus normal samples.

**Table 1:** Significant Changes of PTPN Expression in Transcription Level between Different Types of Pancreatic Cancer and Normal Tissues (Oncomine Database).

	Type of Pancreatic Cancer versus Normal Pancreat c Tissue	Fold Change	p Value	t Test	Source and/or Reference
PTPN1	Pancreatic Ductal Adenocarcinoma Epithelia	1.542	0.040	1.850	Grutzmann Pancreas
PTPN2	Pancreatic Ductal Adenocarcinoma	1.895	0.024	2.044	Ishikawa Pancreas
PTPN4	Pancreatic Ductal Adenocarcinoma	1.625	0.028	1.964	Ishikawa Pancreas
PTPN6	Pancreatic Adenocarcinoma	1.754	1.74E-04	4.696	lacobuzio--Donahue Pancreas 2
PTPN9	Pancreatic Adenocarcinoma	2.229	6.06E-04	5.526	lacobuzio-Donahue Pancreas 2
	Pancreatic Ductal Adenocarcinoma	1.664	0.019	2.232	Grutzmann Pancreas
	Pancreatitis	2.697	0.004	4.488	Logsdon Pancreas
PTPN11	Pancreatic Ductal Adenocarcinoma	1.602	0.048	1.708	Ishikawa Pancreas
	Pancreatic Ductal Adenocarcinoma	2.122	0.040	1.641	Grutzmann Pancreas
PTPN12	Pancreatic Adenocarcinoma	4.631	1.60E-05	8.465	Logsdon Pancreas
	Pancreatic Adenocarcinoma	2.443	7.32E-04	5.060	lacobuzio-Donahue Pancreas 2
	Pancreatic Carcinoma	2.448	1.00E-03	3.561	Segara Pancreas
	Pancreatic Carcinoma	2.210	1.05E-04	4.391	PeiPancreas
	Pancreatic Ductal Adenocarcinoma	2.817	1.52E-09	7.348	Badea Pancreas
	Pancreatitis	3.619	2.72E-04	5.613	Logsdon Pancreas
PTPN13	Pancreatic Ductal Adenocarcinoma	2.100	2.99E-09	6.749	Badea Pancreas
PTPN22	Pancreatic Adenocarcinoma	2.770	3.00E-03	3.446	lacobuzio-.Donahue Pancreas 2
	Pancreatic DuctalAdenocarcinoma	126.456	9.18E-04	4.521	Buchholz Pancreas
	Pancreatic lntraepithelial Neoplasia	4.571	9.00E-03	2.506	Buchholz Pancreas
PTPN23	Pancreatic lntraepithelial Neoplasia	1.657	4.20E-02	1.797	Buchholz Pancreas Statistics

In Logsdon dataset, we found that *PTPN12* expressed significantly higher with a fold change of 4.631 in PAAD, and a fold change of 3.619 in pancreatitis versus normal samples. Iacobuzio-Donahue dataset suggested that *PTPN12* also expressed higher in PAAD with a fold change of 2.443 versus normal samples. Besides that, Segara dataset and Pei dataset reported that the increased fold change of *PTPN12* in pancreatic carcinoma versus normal samples are 2.448 and 2.210, respectively. Furthermore, we found that *PTPN12* overexpressed in pancreatic ductal adenocarcinoma with a fold change of 2.817 in Badea dataset. The expression of *PTPN13* was also higher in pancreatic ductal adenocarcinoma with a fold change of 2.100 in Badea dataset versus normal samples. In Iacobuzio-Donahue dataset, we found that *PTPN22* expressed higher in PAAD (fold change = 2.770), and, in Buchholz data (**Table 1**), *PTPN22* expressed higher in pancreatic ductal adenocarcinoma (fold change = 126.456), pancreatic intraepithelial neoplasia (fold change = 4.571) versus normal samples. Additionally, in Buchholz Pancreas Statistics dataset, we found that *PTPN23* expressed higher in pancreatic intraepithelial neoplasia (fold change = 1.657) compared to normal samples (**Table 1**). In summary, the results suggested that the expression status of *PTPN1/2/4/6/9/11/12/13/22/23* were upregulated in PAAD.

### Relationship Between PTPN mRNA Levels and Clinicopathological Parameters in Pancreatic Adenocarcinoma Patients

**Figure 2:**
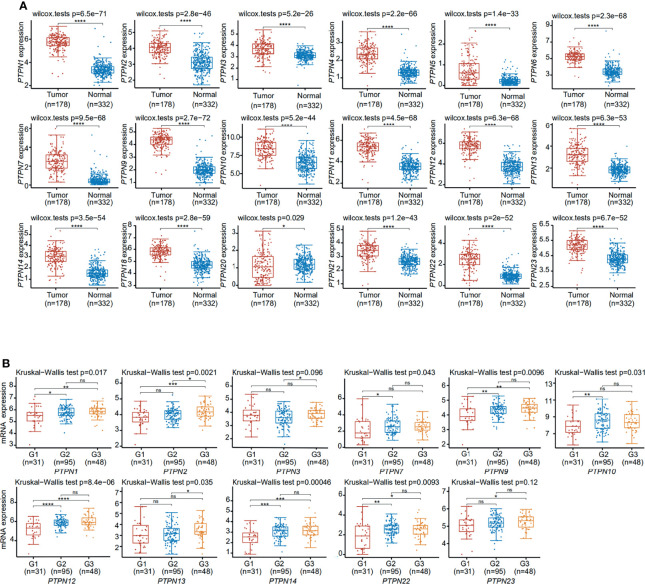
Expression of PTPNs in pancreatic adenocarcinoma. **(A)** The differentiation and expression of PTPN in tumor and adjacent tissues. **(B)** The significant differential expression of PTPNs at different grades. ns, No Significant. *p < 0.05, **p < 0.01, ***p < 0.001, ****p < 0.0001.

To explore the expression of PTPN family members in PAAD, we compared PTPNs mRNA expression in pancreatic tumor and pancreatic tissue from The Cancer Genome Atlas (TCGA) dataset (https://portal.gdc.cancer.gov/). This study has shown that PTPNs expression levels in normal tissues are lower than those in PAAD tissues (**Figure 2A**). And we analyzed PTPNs expression levels in the grades of PAAD. *PTPN1*, *PTPN2*, *PTPN7*, *PTPN9*, *PTPN10*, *PTPN12*, *PTPN13*, *PTPN14*, and *PTPN22* groups significantly varied, whereas *PTPN3*, *PTPN4*, *PTPN5*, *PTPN6*, *PTPN11*, *PTPN18*, *PTPN20*, *PTPN21*, and *PTPN23* groups did not significantly differ (**Figure 2B** and **Supplementary Figure S1**). Among them, *PTPN2* is a more significant one.

**Figure 3:**
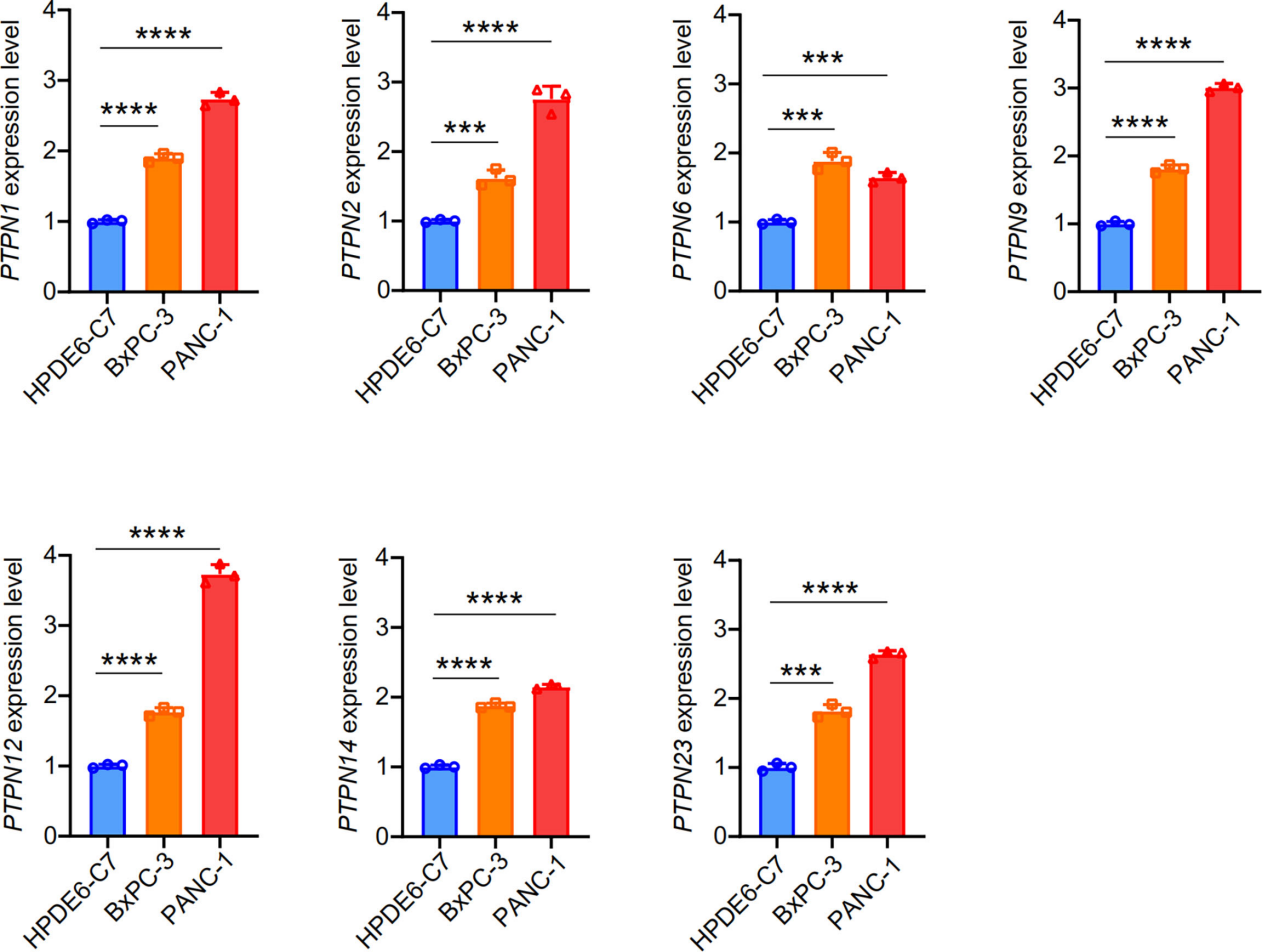
Expression of PTPNs in HPDE6-C7, BXPC-3 and PANC-1 cells. The results from three independent experiments were statistically analyzed using t-test method. ***p < 0.001, ****p < 0.0001.

Subsequently, we detected the expression of some significantly different PTPN family genes in PANC-1, BXPC-3, and HPDE6-C7 through RT-qPCR (**Figure 3**). The results showed that the expressions of *PTPN1/2/6/9/12/14/23* in PAAD cells were significantly up-regulated than those in normal pancreatic cells.

### Relationship Between Elevated Expression of *PTPN2* and *PTPN12* mRNA and Prognosis in Patients With Pancreatic Adenocarcinoma

**Figure 4:**
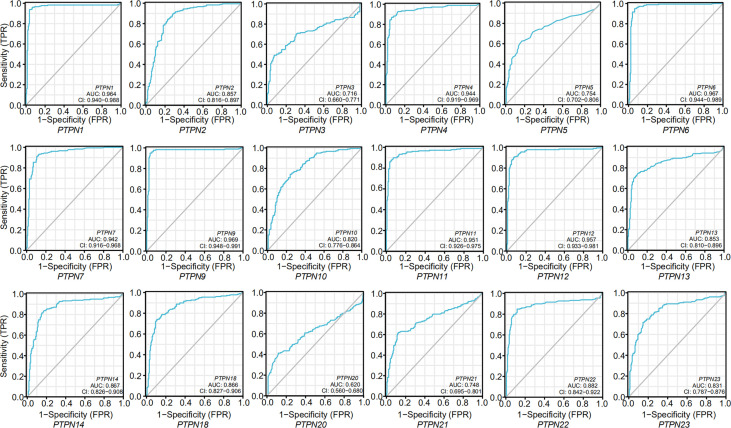
ROC curves for the relationship of PTPNs expression and the malignant progression of pancreatic adenocarcinoma.

After we confirmed PTPNs expression in PAAD stages, and we analyzed Receiver Operating Characteristic (ROC) curves to explore the effect of PTPN expression on the deterioration of PAAD (**Figure 4**). The area under the curve (AUC) values of the ROC curves of *PTPN1/2/6/7/9/11/12/13/14/18/22* were at the range of 0.85 to 0.99, which predicted that the expressions of these PTPNs might be related to the progression of PAAD.

**Figure 5:**
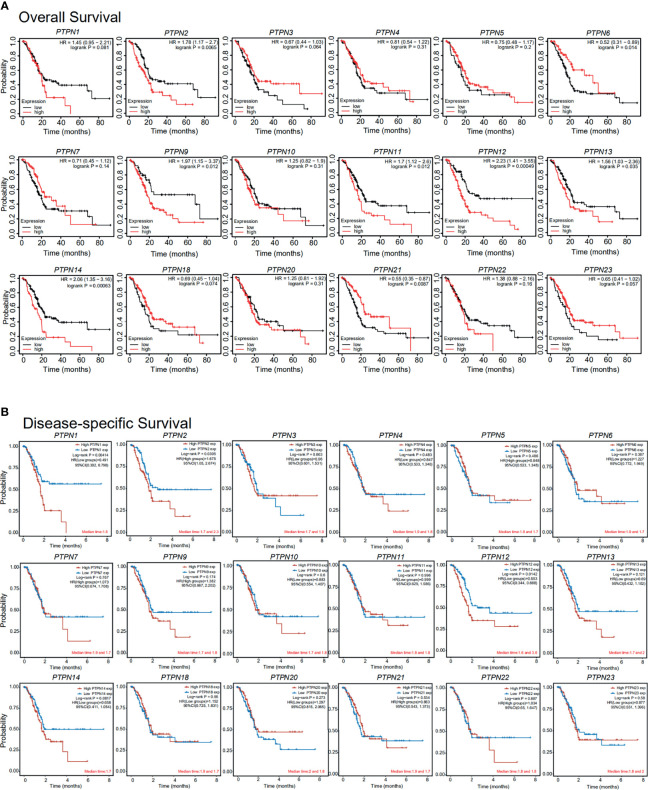
Importance of the mRNA level of PTPNs in the prognosis of patients with pancreatic adenocarcinoma (Kaplan-Meier plot). *p < 0.05, **p < 0.01. HR: Hazard ratios, 95%CI: 95% Confidence interval.

To examine the key role of PTPNs in the survival of PAAD patients, we analyzed the linkage of PTPNs mRNA levels and survival rates using Kaplan-Meier Mapping Tool and public databases. As shown in **Figure 5A**, this analysis also showed that the *PTPN2*, *PTPN9*, *PTPN11*, *PTPN12*, *PTPN13*, *PTPN14* mRNA levels increased and the *PTPN6*, *PTPN21* mRNA levels decreased, which were significantly correlated with the overall survival (OS) (p < 0.05). And the results also showed that elevated levels of *PTPN1*, *PTPN2*, and *PTPN12* mRNA in PAAD patients were significantly associated with disease-specific survival (DSS) (p < 0.05, **Figure 5B**). PAAD patients which have high *PTPN2* and *PTPN12* mRNA expression are predicted to have poor OS and DSS.

### High *PTPN2* Expression Was Independent Predictive Factors for Poor Outcomes in Pancreatic Adenocarcinoma Patients

Recently, *PTPN2* has been identified as a potential target for cancer treatment. Many researches have revealed that the absence of *PTPN2* can potentiate anti-tumor immune effects (20, 22, 25, 31). Loss of functional variants in the *PTPN2* gene encoding T cell tyrosine phosphatase (TCPTP) is connected with an increasing risk of chronic inflammatory diseases, including rheumatoid arthritis (32) and type I diabetes (33), Celiac disease and IBD, ulcerative colitis, and Crohn’s disease (33, 34). However, the function of *PTPN2* in PAAD has not been reported. Here, preliminary analyzes showed that *PTPN2* expression level correlates positively with the malignant progression of PAAD.

**Figure 6:**
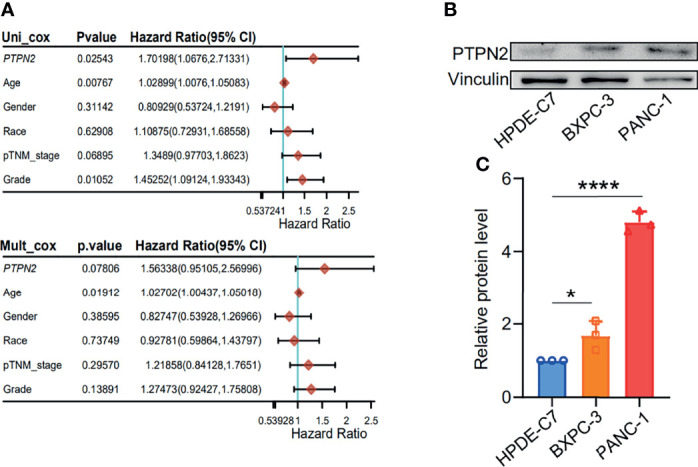
Increased *PTPN2* expression is associated with the prognosis of pancreatic adenocarcinoma. **(A)** Univariate regression analysis of clinical information in TCGA PAAD samples. **(B)** The protein levels of PTPN2 were detected by western blot analysis in PAAD cells. vinculin was used as an endogenous control. **(C)** Grayscale analysis of the western blot results. *p < 0.05, ****p < 0.0001.

For patients with PAAD, Univariate Cox regression analysis demonstrated that high *PTPN2* mRNA level (P < 0.05), Age (P = 0.00767), Grade (P < 0.05) were independent prognostic factors (**Figure 6A**). In contrast, Race, Gender, and TNM-stage were not independent prognostic factors (**Figure 6A**).

### Down-Regulation of *PTPN2* Significantly Reduces the Burden of Pancreatic Adenocarcinoma

**Figure 7:**
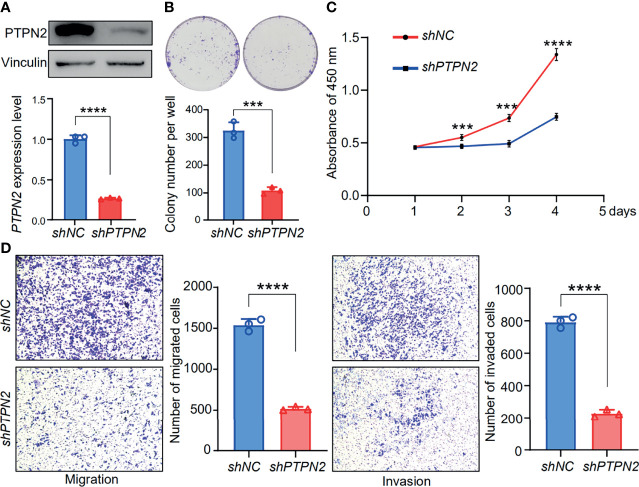
Functional analysis of *PTPN2* knock down in pancreatic adenocarcinoma cells. **(A)** The protein levels of PTPN2 were detected by RT-qPCR and western blot analysis in shPTPN2 or vector-transfected PANC-1 cells. vinculin was used as an endogenous control. (B, C and D) Effects of knock-down of *PTPN2* on the colony formation **(B)**, cell growth **(C)**, migration and invasion **(D)** in PANC-1 cell lines. ***p < 0.001, ****p < 0.0001.

**Figure 8:**
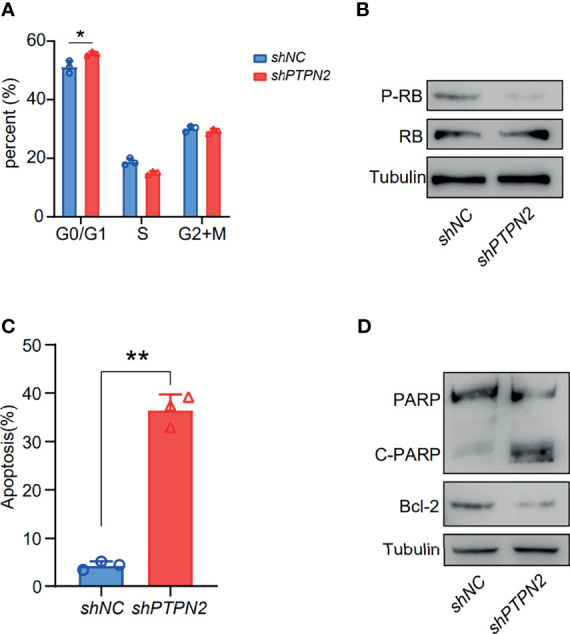
Depletion of *PTPN2* induction of cell cycle arrest and apoptosis. **(A)** Cell cycle phase distribution of PANC-1 *PTPN2* KD cells determined by flow cytometry. **(B)** Western blotting analysis of cell cycle related protein changes upon knock-down of *PTPN2*. **(C)** Representative flow cytometry plot for quantifying apoptosis in PANC-1 *PTPN2* KD and control cells, analyzed by 7-AAD and Annexin V staining. **(D)** Western blotting analysis of apoptosis related protein changes upon knock-down of *PTPN2*. *p < 0.05, **p < 0.01.

We tested the mRNA levels of *PTPN2* in three different cell lines for studying the role of PTPN2 in PAAD. The *PTPN2* mRNA level of PANC-1 and BXPC-3 cell lines was markedly up-regulated compared to normal HPDE6-C7 cell lines (**Figure 3**). Subsequently, we conducted western blot (WB) analysis to evaluate protein expression levels of PTPN2 in BXPC-3, HPDE6-C7, and PANC-1 cell lines. The analysis showed that *PTPN2* expression was up-regulated in PANC-1 and BXPC-3 cell lines than that in HPDE6-C7 cell lines (**Figures 6B, C**). Therefore, we selected PANC-1 with the highest *PTPN2* expression for further experiments. We use shRNA to deplete *PTPN2* in PANC-1 cell line (**Figure 7A**). *PTPN2* depletion significantly suppressed the cell cloning (**Figure 7B**), the cell growth (**Figure 7C**), migration and invasion in PANC-1 cell line (**Figure 7D**). In addition, knock-down of *PTPN2* in PANC-1 cells significantly induced cell cycle arrest and apoptosis (**Figures 8A, C**). BCL-2 and cleaved-PARP, which are cell apoptosis-related proteins, were down-regulated and up-regulated respectively. In the *PTPN2*-depleted PANC-1 cells line we detected the expression of P-RB decreased, which illustrated that cell cycle arrest in the G1 phase (**Figures 8B, D**). In summary, reducing the level of *PTPN2* in cells can markedly reduce the malignancy of PAAD, suggesting that *PTPN2* plays critical roles in regulating PAAD and is a potential target to develop novel anti-cancer drugs.

### Mechanistic Investigation Into How *PTPN2* Promotes Pancreatic Adenocarcinoma Proliferation

**Figure 9:**
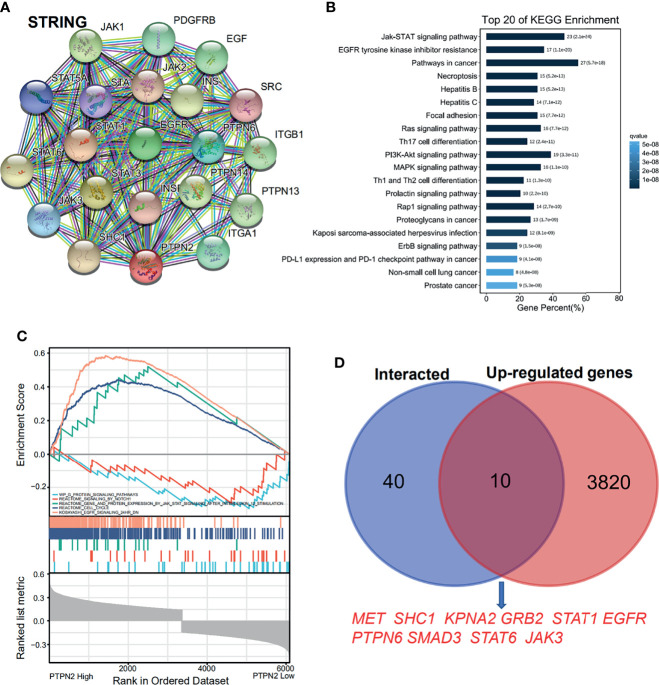
Proteins that interact with *PTPN2* and *PTPN2* related pathway enrichment in pancreatic adenocarcinoma. **(A)** The PPI analysis of PTPN2 (STRING). **(B)** KEGG enrichment of the PPI genes. **(C)** GSEA enrichment of the *PTPN2* correlated genes. **(D)** online prediction of the common genes in PTPN2 PPI network and PTPN2 positively correlated genes.

The PAAD samples collected by TCGA were separated into high *PTPN2* expression and low *PTPN2* expression groups to investigate the importance of the two different functional sets. In order to clarify how *PTPN2* regulates PAAD, we used the web-based software system EMTome to analyze the role of *PTPN2* in PAAD patients. The heat map showed that 3,174 genes in PAAD have a negative correlation with the *PTPN2* expression, and 3,830 genes have a positive correlation with the expression of *PTPN2* in pancreas cancer (**Supplementary Figure S2A**). 59 proteins that could bind to PTPN2 were characterized by a STRING interactive network (**Figure 9A**). Using the Omicsmart online platform, 59 genes were enriched through the KEGG pathway. The main pathways correlated with 59 genes are JAK-STAT signaling pathway, EGFR tyrosine kinase inhibitor resistance and cancer pathways (**Figure 9B**). Through Gene Set Enrichment Analysis (GSEA) analysis, the high *PTPN2* expression was found to be significantly associated with the JAK-STAT signaling pathway (**Figure 9C**). Ten genes appeared in both the top 50 PPI network-related genes of *PTPN2* and the positively correlated genes (**Figure 9D**) and six genes appeared in both the top 50 PPI network-related genes of *PTPN2* and the negatively correlated genes (**Supplementary Figure S2B**).

**Figure 10:**
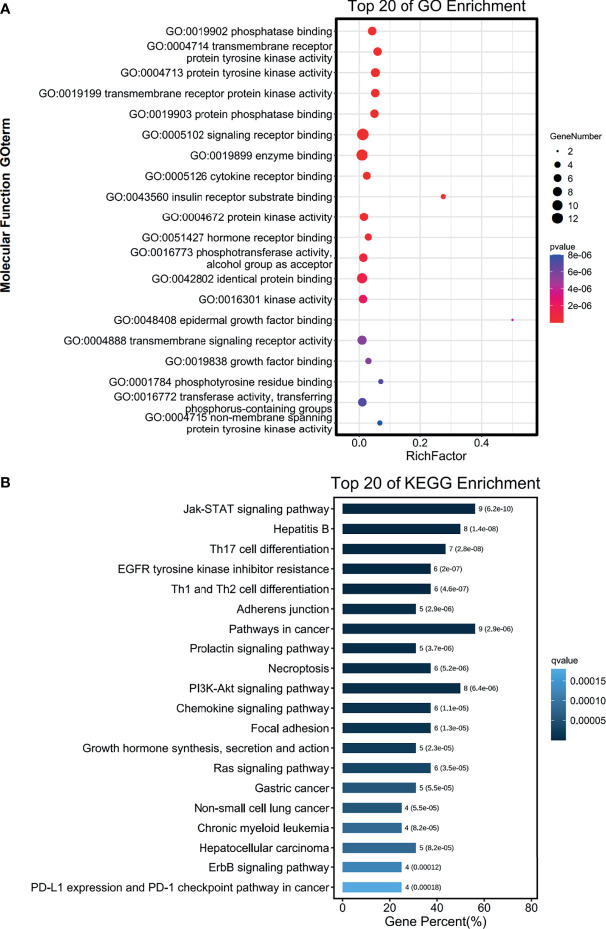
Functions of the genes significantly associated with *PTPN2*. **(A, B)** The functions of common genes in PTPN2 PPI network and PTPN2 correlated genes were predicted by the analysis of gene ontology (GO) **(A)** and Kyoto Encyclopedia of Genes and Genomes (KEGG) **(B)**.

**Figure 11:**
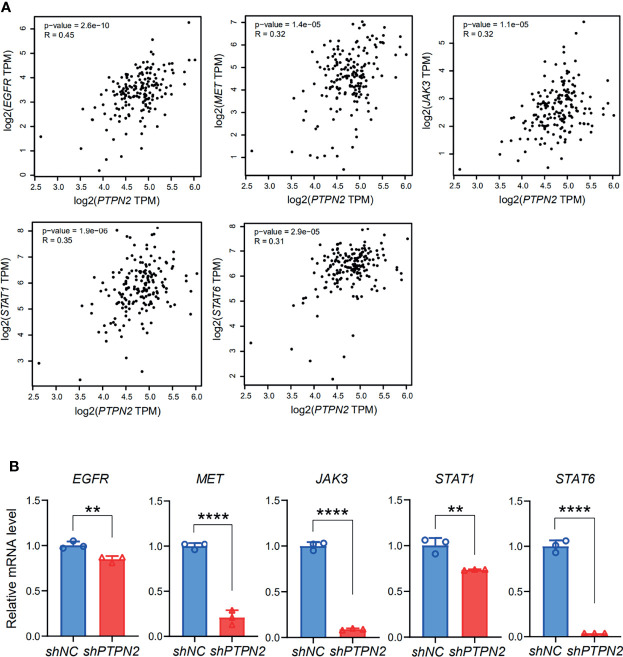
Identification of a series of targets of *PTPN2*. **(A)** Correlation analysis of *PTPN2* expression and key genes expression in PAAD. **(B)** RT-qPCR analyze the relative expression of *EGFR*, *MET*, *STAT1*, *JAK3 and STAT6* in sh*PTPN2* or vector-transfected PANC-1 cells. **p < 0.01, ****p < 0.0001.

The functions of the sixteen genes were enriched through analyzing GO and KEGG databases. GO enrichment analysis predicts genes function from different aspects of biological process, cell composition and molecular function. We found that GO:0004715 (non-membrane spanning protein tyrosine kinase activity), GO:00167772 (transferase activity, transferring phosphorus-containing groups) and GO:0001784 (phosphor-tyrosine residue binding) were markedly enriched by the sixteen genes in PAAD (**Figure 10A**). These sixteen genes alterations also control GO:0060397 (JAK-STAT cascade involved) and GO:0070435 (Sch-EGFR complex) markedly (**Supplementary Figures S3A, B**). They are well-known genes involved in the JAK-STAT signaling pathway. These sixteen genes were enriched by KEGG pathway using Omicsmart online platform. The main pathways were JAK-STAT signaling pathway, hepatitis B and Th17 cell differentiation (**Figure 10B**). Correlation analysis by GEPIA2 demonstrated that the transcriptional level of *EGFR*, *JAK3*, *STAT1*, *STAT6*, *MET* are significantly associated with *PTPN2* (**Figure 11A**), then, we used RT-qPCR to analyze the expression of these genes under different conditions, and silenced *PTPN2* expression in PANC-1 cells, the expression of these genes was markedly reduced (**Figure 11B**). It proved that these genes are involved in *PTPN2*-regulated signaling pathway.

### Immune Infiltration and Immunotherapy Related to *PTPN2* Expression

**Figure 12:**
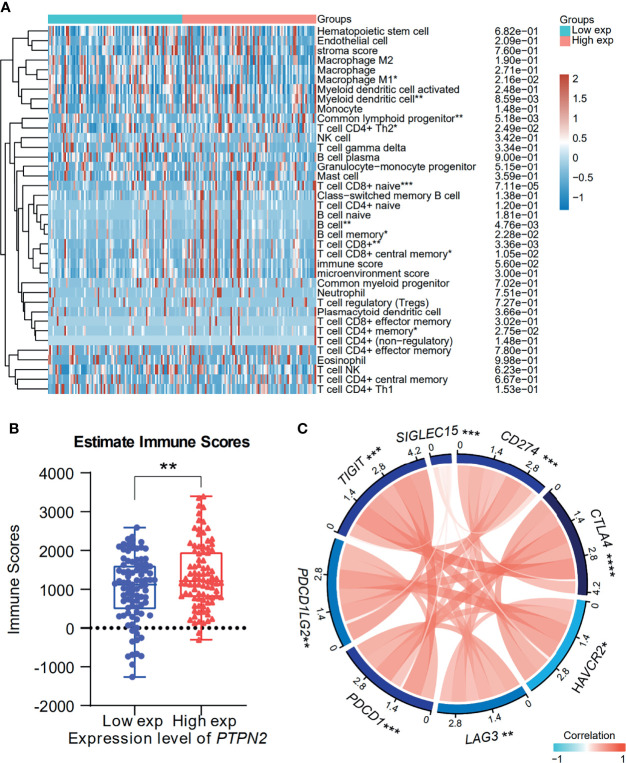
Correlation analysis of *PTPN2* expression with TIICs and Immune Score in pancreatic adenocarcinoma. **(A)** Immune cells score heatmap associated with *PTPN2* expression. **(B)** Comparison of *PTPN2* expression between the high and low immune score groups of PAAD. **(C)** Correlation analysis of *PTPN2* expression levels with 8 immune checkpoint gene levels in PAAD. *p < 0.05, **p < 0.01, ***p < 0.001, ****p < 0.0001.

The degree of tumor invasion by tumor immune infiltrating cells (TIICs) is an important part of the tumor environment, which exhibits an important influence in tumor prognosis (35). Therefore, we investigated the correlation between *PTPN2* expression and TIICs in PAAD. The results showed that high PTPN2 expression is significantly related to B cells, CD8^+^ T cell and low *PTPN2* expression is significantly related to macrophage M1(**Figure 12A**). Then, we downloaded the immune score of PAAD patients from TCGA to evaluate the extent of immune cell infiltration in the tissue. The results indicated that patients with high immune scores had higher *PTPN2* expression (p < 0.01, **Figure 12B**). Immune checkpoint inhibitors (ICIs) are a novel tumor immunotherapy strategy, which can significantly improve the prognosis of many cancer patients (36). Then, we further detected the relationship between *PTPN2* expression and the expression of 8 immune checkpoint molecules. Interestingly, in PAAD, *PTPN2* expression was associated with these immune checkpoint markers, including *CD274 (PD-L1)*, *CTLA4*, *HAVCR2*, *LAG3*, *PDCD1*, *PDCD1LG2*, *TIGIT and SIGLEC15* (**Figure 12C**). Therefore, these results strongly prove that *PTPN2* gene may exhibit a critical role in tumor immunotherapy.

## Discussion

PAAD is still a deadly malignant tumor. It is not only the sixth leading cause of cancer death in China but also the fourth leading cause of cancer death in the West (37, 38). Compared to other cancers, PAAD has a high degree of malignancy, low resection rate, high recurrence rate, and poor prognosis. Although new treatments are being studied, including immunotherapy and targeted therapy, most drugs have not produced satisfactory results (39, 40). Therefore, the identification of novel biologic therapeutic targets is remarkable and urgent for PAAD.

PTPNs is a momentous member of protein tyrosine phosphatase, and together with protein tyrosine kinase, it regulates the phosphorylation and dephosphorylation of tyrosine in cell signal transduction (17). Currently, various studies have focused on the relationship between the PTPNs and various kinds of cancer. We used Oncomine and TCGA online database to study the expression pattern of PAAD PTPN genes, and further establish ROC model and test its diagnostic value. We conducted human cells mRNA expression experiments by RT-qPCR to validate our results. Our study found that all members of the PTPN family have high levels of expression in human PAAD. PTPNs might be developed as a diagnostic biomarker for PAAD. And previous researches have shown that *PTPN1*, acting as an oncogene, inhibits the activation of AMPK by PKM2 and reduces the level of phosphorylation of PRAS40, thereby inhibiting mTOC1. The inhibition of mTOC1 and p70S6K can lead to a proliferation arrest of PAAD cells (23). In our research, the Oncomine dataset and TCGA dataset show that *PTP1B* expression in normal tissue is down-regulated in PAAD. *PTP1B* mRNA expression level in normal pancreatic cells is significantly lower than that in PAAD cells. We determined the prognostic value of *PTP1B* in patients, who get PAAD, by the Kaplan-Meier plotter. In human CRC tissues, the expression and activity of the *PTPN2* protein are greatly increased and immune cells are significantly increased (21). The high *PTPN2* expression is correlated with the decrease in T cell activity, recruitment and cytotoxicity (41). We report that *PTPN2* expression in PAAD tissues is much higher than that in normal tissues. *PTPN2* expression in patients who get PAAD is significantly correlated with tumor stage. Especially for PAAD cells, the *PTPN2* mRNA level is much higher than in normal pancreatic cells. A high *PTPN2* expression was significantly correlated with poor OS, DSS in PAAD patients. In bile cancer, *PTPN3* is an oncoprotein that can stimulate cell proliferation, invasion and poor prognosis (42). *PTPN4* is a tumor suppressor that dephosphorylates the Tyr705 residue from p-STAT3 instead of Ser727, resulting in the inhibition of the transcriptional activity of *STAT3* (43). The activation of *PTPN5* inhibits the MAPK signaling pathway induced by EGF in breast cancer cells. In the *p53* mutant TNBC xenograft mouse model, the activation of *PTPN5* can effectively inhibit the deterioration of breast cancer, indicating *PTPN5* could be a breast cancer tumor suppressor (44). In our research, the results indicated that *PTPN3*, *PTPN4*, *PTPN5* expression in normal tissues was lower than in PAAD tissues, but the tumor stage of PAAD patients did not affect the expression. It was not statistically significant that lower *PTPN3*, *PTPN4*, *PTPN5* expressions were correlated with poor OS, DSS in all patients with PAAD. It is known that *PTPN6* (*SHP-1*) can modulate intracellular signals from a variety of transmembrane receptors. The decreasing of *SHP-1* expression and activity leads to increase the activity of JAK kinase, which can cause the growth of abnormal cells (45). In our study, the *PTPN6* expression in normal tissues is lower when compared to pancreatic tumor tissues. *PTPN6* mRNA expression level in normal pancreatic cells is markedly lower than that in PAAD cells. It suggested that *PTPN6* could serve as a diagnostic biomarker of PAAD by ROC results. Interestingly, in PAAD patients, low PTPN6 expression was significantly correlated with poor OS. *PTPN7* (also known as hematopoietic PTP) is a cytoplasmic protein tyrosine phosphatase which is cloned from T lymphocytes of human originally. T cells which are originated from *PTPN7*-KO mice showed ERK hyperphosphorylation, indicating that *PTPN7* dephosphorylated ERK, thereby down-regulating T cell activation (46). In our study, we confirmed that *PTPN7* expression in normal tissue is lower than in PAAD tissue, However, the correlation between this expression and tumor stage was not obvious in PAAD patients. In PAAD patients, higher *PTPN7* expressions were correlated with poor OS, DSS, but it was not statistically significant. *PTPN9* is a cytoplasmic PTPs widely distributed in brain tissue, white blood cells, endocrine cells and other tissues (47). *PTPN12* plays a critical role in tumor deterioration of TNBC (48), and *PTPN12* is an independent prognostic factor for hepatocellular carcinoma (HCC) (49). *PTPN14* acts as a possible tumor suppressor, especially, studies have shown that *PTPN14* interacts with *YAP1* and ultimately inhibits cell proliferation, and promotes the density-dependent cytoplasmic translocation of *YAP1* (50). We report that the expression of *PTPN9*, *PTPN12* and *PTPN14* in normal tissues is lower than that in PAAD tissues. *PTPN9, PTPN12* and *PTPN14* mRNA expression levels in normal pancreatic cells are markedly lower than those in PAAD cells. The ROC results show that *PTPN9*, *PTPN12* and *PTPN14* could be used to be diagnostic markers of PAAD. And higher the expression of *PTPN9*, *PTPN12*, *PTPN14* was markedly correlated with poor OS in patients with PAAD. *PTPN10* (*DUSP1*) is an anti-apoptotic phosphatase, which is expressed in various tissues, with the highest content in the heart, lung and liver (51). Targeting *PTPN22* offers the potential to improve cancer immunotherapy using at least two clinically proven therapies and signaling pathways: IFNAR and TCR signaling pathways. Since *PTPN22* plays an important role in other signaling pathways, other mechanisms may further enhance tumor immunity (29). The deterioration of liver cancer can be achieved by overexpression of *PTPN11* (26). In our research, we confirmed that *PTPN10* and *PTPN22* expression in normal tissues is lower than that in PAAD tissues, and this expression is correlated with the tumor stage of patients with PAAD. And higher *PTPN10* and *PTPN22* expression in patients with PAAD is associated with poorer OS and DSS, but there was no statistical significance. The previous article studied the role of *PTPN13* in FAS-mediated apoptosis. The interaction of *PTPN13* and the FAS (CD95/APO-1) death receptor is reported as the first evidence (52). In our report, the results showed that *PTPN13* expression in normal tissues is lower than that in PAAD tissues, but *PTPN13* expression in PAAD patients has nothing related to the tumor stage. In PAAD patients, higher *PTPN13* expressions were markedly related to poor OS, but DSS was not statistically significant. Recent studies have shown that knocking down *PTPN18* may inhibit tumor proliferation in endometrial cancer and stimulate cell apoptosis (53). *PTPN21* is highly expressed in hematopoietic stem cells, after the deletion, the retention of hematopoietic stem cells in the niche is impaired, resulting in defects in the hematopoietic function (54). Our research also proved that *PTPN18*, *PTPN21* expression in normal tissues was lower than that in PAAD tissues. However, in PAAD patients, this expression was not correlated with the tumor stage. It was not statistically significant that higher *PTPN18* expressions were correlated with poor OS, DSS in PAAD patients. Yet, lower expressions of *PTPN21* were significantly related to poor OS in PAAD patients. Many physiological functions of *PTPN23* may be related to the activity of this tumor suppressor, suggesting many mechanisms. Many organizations often report that the lack of *PTPN23* is a factor of cell adhesion, migration and invasion, which is closely related to the tendency of metastasis (55). In our report, *PTPN23* expression in normal tissues is lower than that in PAAD tissues. And *PTPN23* expression level mRNA in normal pancreatic cells is much higher than that in PAAD cells. Interestingly, lower *PTPN23* expressions were correlated with poor OS, DSS in PAAD patients, but the relationship was not statistically significant.

Although the PTPN family has different mechanisms of action in many kinds of diseases, there are not much researches on the relationship between PTPN family members and pancreatic cancer (56). Our study examines the mRNA expression and the prognostic value (OS, DSS) of various PTPN factors in PAAD for the first time. We hope to enrich existing knowledge and improve the treatment plan by our findings. Among these PTPN family members, we selected *PTPN2* for further analysis because of the strongest effect of *PTPN2* on the malignant of PAAD.

After analysis, in PAAD, we found that 3,830 genes were positively correlated with *PTPN2* expression. Among them, there were 59 genes involved in *PTPN2* PPI network. After systematic analysis, we found that *STAT1* and *EGFR* were key factors which regulate *PTPN2* in the deterioration of PAAD. *STAT1* and *EGFR* expression in normal were lower than in PAAD and positively correlated with *PTPN2* expression. *EGFR* is important for cell proliferation and migration. However, its abnormal activity in the pathogenesis of human cancer requires an understanding of the complex regulation of *EGFR* activity and downstream signaling events. The mRNA expression level of *EGFR* was positively correlated with *PTPN2* in PAAD. Knocking down *PTPN2* induced the repression of *EGFR*. *PTPN2* induced the expression of *EGFR* to promote PAAD. *STAT1* regulates cell death *via* transcription-dependent and transcription-independent mechanisms. *STAT1* directly regulate and control several genes expression, such as *PARP* and *BCL-2*. Repression of *PTPN2* inhibited the expression of *STAT1*. Taken together, *PTPN2* can regulate the expression of *STAT1* and *EGFR*, therefore, *PTPN2* is closely related to PAAD. Moreover, the high expression of *PTPN2* promotes the infiltration of PAAD immune cells and is positively correlated with immune checkpoints expression, suggesting that patients with high expression of *PTPN2* could respond better to immune checkpoint blocking therapy.

*PTPNs/PTPN2* can not only affect the growth of the PAAD, but also promote PAAD cells infiltration, affect the expression of immune checkpoints, and increase tumor response to immunotherapy. In this study, we discussed the critical roles of *PTPN2* in regulating PAAD, which could be used as a valuable target to design and develop effective small molecule anti-cancer drugs. In addition, studies have shown that *PTPN2* deficiency might enhance the therapeutic effect of CAR-T cells in malignant tumors, therefore, we can combine *PTPN2* small molecule inhibitors with immunotherapy drugs to obtain a better therapeutic effect on tumors.

## Conclusion

In summary, we analyzed the influence of PTPN family members on PAAD. The PTPN family members in normal tissues are lower than those in PAAD tissues. The Receiver Operating Characteristic (ROC) curves show that *PTPN1/2/6/7/9/11/12/13/14/18/22* might be related to the progression of PAAD. Among these members, *PTPN2* was the most important factor that regulated the progression of PAAD by regulating JAK-STAT signaling pathway. Furthermore, *PTPN2* high expression facilitated the infiltration of the immune cells in PAAD and showed a strong positive correlation with immune checkpoints expression, which implies that higher *PTPN2* level patients may improve response to the blockage treatment of immune checkpoints. Taken together, our findings suggested that *PTPN2* could be used as a new biomarker for the prognosis and a potential target to develop novel treatment of PAAD patients.

## Materials and Method

### PTPNs Expression Analysis in Pan-Cancer by Oncomine Database

We use Oncomine online cancer gene expression data website (www.oncommine.ORG) to analyze microarray information and a set of bioinformatic data. The mRNA expression of PTPNs in cancer samples was compared with samples in normal from Oncomine database, and the p value was acquired by Student t-test. P values < 0.05 and folding change < 1.5 were supposed to be statistically significant.

### PTPNs Expression Analysis in Pancreatic Adenocarcinoma

Download tumor RNA sequence data using the genome data sharing (GDC) data portal (https://portal.gdc.cancer.gov/), including expression data of 178 tumor tissues and the mRNA expression data of 4 pairs of normal tissue samples. We obtained the data of 328 normal tissue samples through GTEX V8 version (https://gtexportal.org/home/datasets). PTPNs expression in tumor and normal tissues was tested by Wilcox test using R software v4.0.3. Then, the mRNA expression data of 31 grade1 samples, 95 grade 2 samples and 48 grade 3 samples in PAAD were downloaded using GDC. Kruskal Wallis one-way ANOVA was performed on each gene of PTPN family using R software v4.0.3. P < 0.05 was supposed to be statistically significant.

### Prognosis and Diagnostic Analysis of PTPN Family Genes in Pancreatic Adenocarcinoma

The effect of PTPN mRNA expression on patient survival was evaluated by Kaplan-Meier Plotter (http://www.kmplot.com/). Divided into a group with high expression and group with low expression according to the median expression. The 95% confidence interval (CI), log rank risk ratio (HR) and P value of overall survival were evaluated.

The Cancer Genome Atlas (TCGA) dataset (https://portal.gdc.com) is the original clinical data source of 178 PAAD RNA sequencing information. The p value of disease-specific survival curve (DSS) and the risk ratio (HR) of 95% confidence interval (CI) were obtained by Cox proportional hazards regression and log rank test. The analysis method is implemented by survival package and survminer package in R software (v4.0.3).

ROC curve and calculated area under curve (AUC) value are analyzed by R package pROC, and visualization is carried out by ggplot2. An AUC value between 0.5 and 0.7 indicates evidence of model success, a value between 0.7 and 0.9 indicates strong evidence of model success, and a value greater than 0.9 indicates strong evidence of model success.

### Cox Regression Analysis of *PTPN2* in Pancreatic Adenocarcinoma

The 178 samples were grouped according to clinical information and pathological parameters, including *PTPN2* expression level, age, gender, race, tumor stage. Univariate and multivariate Cox regression constructed by the survival package (Cox) in R software was applied to estimate the correlation between PTPN2 expression and PAAD.

### Analysis of Genes Differentially Expressed With *PTPN2* in Pancreatic Adenocarcinoma

Linkedomics (http://www.linkedomics.org/login.php) was used to detect *PTPN2* expression related genes in PAAD. The up-regulated and down-regulated genes were analyzed respectively and visualized by heat map. Pearson’s test was used to evaluate the significant correlation between genes.

### Gene Set Enrichment Analysis (GSEA)

By downloading and screening the genes with different expressions from *PTPN2* gene in PAAD from linkedomics, GSEA enrichment analysis was carried out. GSEA analysis was done using the clusterprofiler R package, and GSEA enrichment plots were drawn using the ggplot2 package in R.Reference gene sets database from Molecular Signatures Database (MSigDB) of C2 (c2.all.v7.2.symbols.gmt).

### Protein-Protein Interaction (PPI) Network Construction

STRING database (http://string-db.org) was employed to predict protein–protein interactions (PPI) and construct the PPI network. It further predicted the protein-protein interaction network of the *PTPN2* gene and observed two main protein clusters that closely interact with each other. Fifty genes were found to directly interact with *PTPN2*, after which they were conducted through KEGG enrichment analysis, 20 of which were used to generate a network diagram.

### GO and KEGG Enrichment Analysis

GO and KEGG enrichment analysis and annotations plotting were performed using the R software package “clusterprofiler”. The 50 genes interacting with PTPN2 protein constructed by PPI network were enriched and analyzed by KEGG. The gene sets with significant positive correlation and negative correlation with *PTPN2* expression screened by linkedomics were intersected with 50 genes with protein interaction respectively. Then, the two intersected gene sets were combined and analyzed by GO and KEGG enrichment, in which, GO analysis included molecular function, biological process as well as cellular component.

### Analysis of Immune-Related Information of *PTPN2*

For reliable immune score evaluation, we used immunedeconv—an R software package in which the integrated Xcell algorithm was used. The heat map of the results is realized by R (v4.0.3) software packages ggplot2 and heatmap.

The 178 PAAD samples downloaded from TCGA were evaluated for the immune scores of the samples using the Estimate package in R software and according to the expression level of *PTPN2*, divide into two groups to create a box plot.

Chord diagram was created in R 3.6.3 by using R software package “circle”. Spearman analysis was promoted on the relationship between PTPN2 gene expression and eight immune checkpoints, and the correlation coefficient and significance relationship were obtained.

### Correlation Analysis Between Screened Key Genes and *PTPN2*

The online tool GEPIA2 (http://gepia2.cancer-pku.cn/) was used to analyze expression correlation between the key genes of interest in the previous intersection genes and *PTPN2* gene.

### Cell Culture

PANC-1 cell line (PAAD) and HPDE6-C7 cell line (normal pancreas) were derived from the American type culture collection center (ATCC). Cell culture was carried out with 10% FBS (fetal bovine serum) without endotoxin DMEM, while BXPC-3 cell line (PAAD) was cultured in RPMI-1640 with 10% FBS.

### Cell Proliferation Assays

5000 PANC-1 cells and PTPN2-deficient PANC-1 cells were seeded in each well of 96 well plate for 5 days. After that, add 10 µl of CCK8 reagent per day to each well of each culture plate, and gently mixed with the cell culture medium. After treatment, the cell culture plate was cultured in an incubator with 5% CO_2_ at 37°C. After four hours, insert the cell culture plate into the enzyme plate analyzer and measure the absorbance at 450 nm.

### Lentivirus Infection

Lentivirus-induced *PTPN2* was modified in PANC-1 cells to obtain *PTPN2* knockdown PANC-1 cells. Lentivirus particles were directly transferred into PANC-1 cells which contain 4 μg/ml polypropylene at 48h and 72h after transfection. Rotate the PANC-1 cell line containing the lentivirus and inoculate it for 90 minutes at 32°C. After rotary inoculation, 2μg/ml puromycin which were used to select positive infected cells were added to the cultured PANC-1 cell. The shRNA sequence: CCGGGATGACCAAGAGATGCTGTTTCTCGAGAAACAGCATCTCTTGGTCATCTTTTTG.

### Real-Time Quantitative PCR (RT-qPCR)

Collect cells cultured according to the above method. The total RNA was separated from cells with TRIZOL reagent, and it was reverse transcribed by using PrimeScripttm RT Kit. The RT-qPCR reaction was carried out using thunderbirdsybr qPCR mixture (QPS201, TOYOBO). Real-time quantitative PCR analysis of gene mRNA expression in cells, standardization by comparing the DDCT method and the GAPDH method. The primers are shown in **Table S1**.

### Western Blot Analysis

The cells were cultured in a T-75 flask with a quantity of 5 x 10^6^/ml, RIPA buffer was used to lyse collected cells. Then, phosphatase inhibitor and protease inhibitor (Roche) were added for mixing, and the protein lysate was obtained after shaking table separation. After quantification by colorimetric protein assay (BioRad), the protein was denatured by boiling in SDS buffer. After size grading by SDS-PAGE, the protein was transferred to PVDF membrane (immobilon). Blocking, antibody culture and washing were carried out in skimmed milk. Dilute the primary antibody against the target protein in the closed solution. The primary antibodies used are Beta Tubulin, Vinculin, PTPN2, BCL-2, PARP, P-RB and RB. The secondary antibody was diluted in a closed solution to detect the species-specific portion of the primary antibody. After washing three times, horseradish peroxidase was incubated with bound secondary antibodies. Electrochemiluminescence (Thermo Fisher Scientific Company) was used for protein detection. Finally, the protein was quantitatively analyzed by Image J software.

### Colony Formation Assays

Place 1,000 cells on a 24-well plate for colony formation analysis. Fix the cells two weeks and stain them with 0.1% crystal violet and count them.

### Migration and Invasion Assays

Transwell plate was used to perform *in vitro* cell migration and invasion experiments on PANC-1 cells and PANC-1 cells transfected with *PTPN2* knockdown. Briefly, cells (2×10^4^ cells per well in 200 μl DMEM without FBS) were plated with 20 μl Matrigel on the upper surface at room temperature seeded into the upper well of the chamber, and the lower well was full of 500 μl DMEM medium containing 10% FBS. After 48 hours of incubation, the cells were fixed in 4% formaldehyde for 30 minutes, stained with Giemsa for 15 minutes and then carefully removed from the top (inside) of the Transwell with a moist cotton swab. Cells that migrated or invaded the bottom surface of Matrigel were counted, and migration was determined by counting the number of cells with a microscope. Three fields were randomly selected for each assay and the average number of migrated cells in three fields was considered as the number of cell migration for the group. For the migration assay, the transwell chambers were not pretreated with Matrigel, but other procedures were performed as described in invasion assays. Three independent experiments were examined, and the groups represent the average of three independent experiments.

### Cell Cycle and Apoptosis Assays

For cell cycle, Collect the cells, wash once with PBS, add 70% ethanol, tap gently, mix for 12 hours at 4°C, centrifuge at 1000g for 5 minutes to pellet. Carefully suck out the supernatant. The kit for cell cycle analysis was stained with propidium iodide (PI) at 37° C for 30 minutes and then used directly for flow cytometry. YF^®^488-Annexin V and PI Apoptosis Kit were chosen to assess Cell apoptosis. Cells were seeded at 1×10^6^/well and then using cold PBS washed cells twice. Transfer 100 μl of the solution to a culture tube and add FITC-Annexin V and PI to each culture tube. Flow cytometry was used to detect the apoptosis assay and FlowJo V10 software was used to analyze the results.

### Statistical Analysis

The results generated in Oncomine are displayed with P-values, fold changes, and ranks. GraphPad Prism 8 (GraphPad Software) was used for statistical analysis. Unpaired Student’s t-tests and Kruskal Wallis one-way ANOVA were used to examine differences between two or more groups. The results of Kaplan-Meier plots and GEPIA2 are displayed with HR and P or Cox P-values from a log-rank test. Spearman correlation analysis was used to evaluate correlations between variables. Univariate and multivariate logistic regression analysis were used to analyze the relationship between *PTPN2* expression levels and clinical characteristics. p values < 0.05 were considered statistically significant.

## Data Availability Statement

The datasets presented in this study can be found in online repositories. The names of the repository/repositories and accession number(s) can be found below: https://www.jianguoyun.com/p/DS6l2oAQrMb_CRiIl5oE.

## Author Contributions

Conception and design: XW, WK, and PY. Acquisition of data (investigation, bioinformatics data mining and process, provided facilities, etc.): JD, WK, XW, JL, MJ, WC, LW, and PY. Analysis and interpretation of data (e.g., statistical analysis, bioinformatics analysis, computational analysis): WK, JD, XW, and PY. Writing-review and editing: WK, JD, XW, and PY. Administrative, technical, or material support: XW and PY. All authors contributed to the article and approved the submitted version.

## Funding

This study was supported by National Natural Science Foundation of China (82073701, 31900687), Natural Science Foundation of Jiangsu Province (BK2019040713) and the Project Program of State Key Laboratory of Natural Medicines, China Pharmaceutical University (SKLNMZZ202013). This study was also supported by Jiangsu Key Laboratory of Drug Design and Optimization, China Pharmaceutical University (No. 2020KFKT-5), and “Double First-Class” University Project (CPU2018GF04).

## Conflict of Interest

The authors declare that the research was conducted in the absence of any commercial or financial relationships that could be construed as a potential conflict of interest.

## Publisher’s Note

All claims expressed in this article are solely those of the authors and do not necessarily represent those of their affiliated organizations, or those of the publisher, the editors and the reviewers. Any product that may be evaluated in this article, or claim that may be made by its manufacturer, is not guaranteed or endorsed by the publisher.
