# Supplementary material for: PTPN2, A Key Predictor of Prognosis for Pancreatic Adenocarcinoma, Significantly Regulates Cell Cycles, Apoptosis, and Metastasis
Source: Front Immunol. 2022 Jan 27;13:805311. doi: 10.3389/fimmu.2022.805311 (PMC8829144; doi:10.3389/fimmu.2022.805311)
Supplement: Supplementary file 1 [file Table_1.docx]

**Supplementary Material**

***PTPN2*, a key predictor of prognosis for pancreatic adenocarcinoma, significantly regulates cell cycles, apoptosis, and metastasis**

Wenbin Kuang^1, 2, #^, Xiao Wang^1, 2, #^, Jiayu Ding^1, 2, #^, Jiaxing Li^1, 2^, Minghui Ji^1, 2^, Weijiao Chen^1, 2^, Liping Wang^1, 2^, Peng Yang^1, 2,^ *

^1^ State Key Laboratory of Natural Medicines and Jiangsu Key Laboratory of Drug Design and Optimization, China Pharmaceutical University, Nanjing 210009, China

^2^ Department of Medicinal Chemistry, School of Pharmacy, China Pharmaceutical University, Nanjing 211198, China

Wenbin Kuang, Xiao Wang, and Jiayu Ding contributed equally to this article

* Corresponding authors: Peng Yang, Email: pengyang@cpu.edu.cn; #639 Longmian Avenue, Jiangning District, Nanjing, 211198, P. R. China. Tel: +86-13681986682;


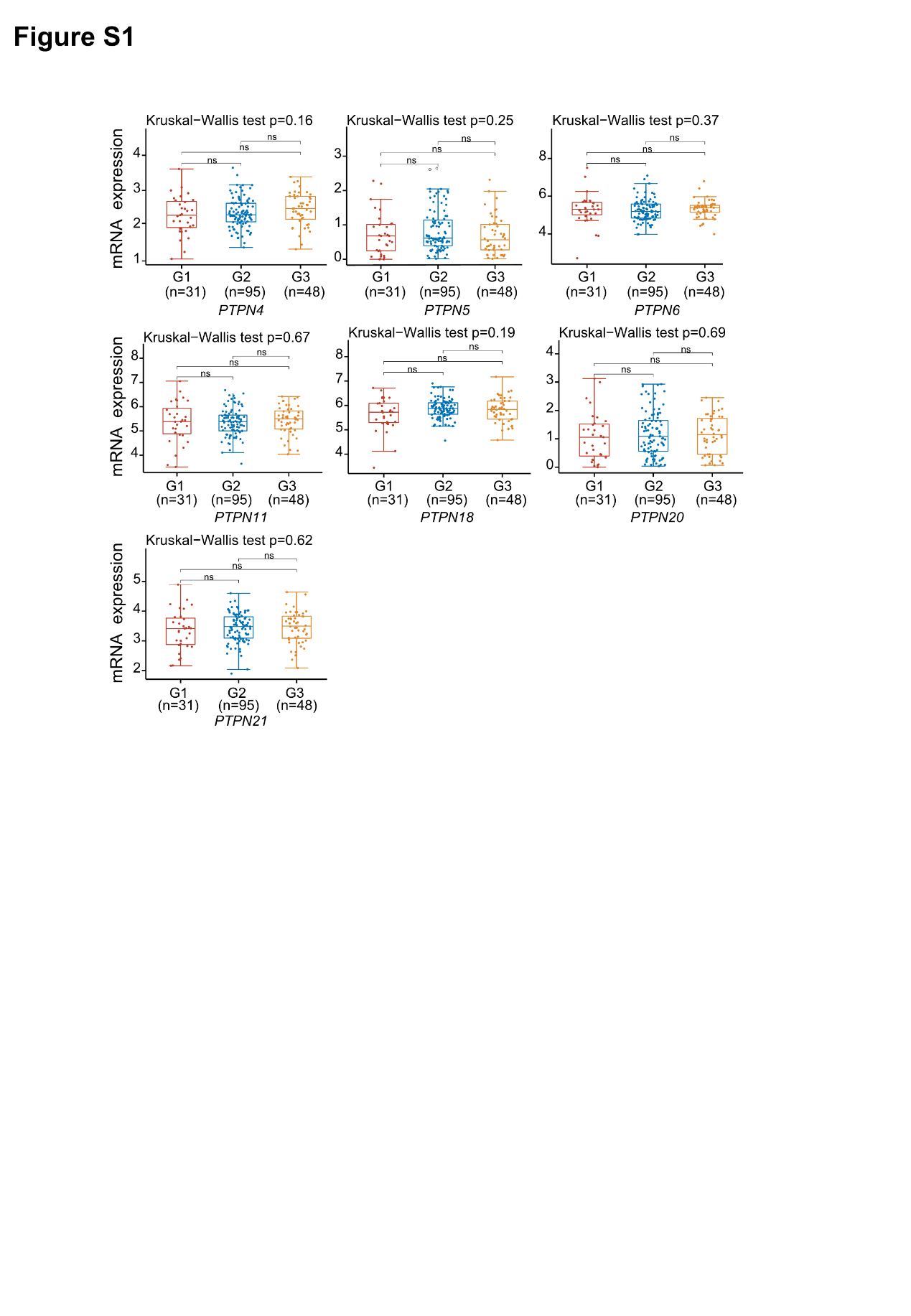


**Supplementary Figure S1. The non-significant differential expression of PTPNs at different grades.**


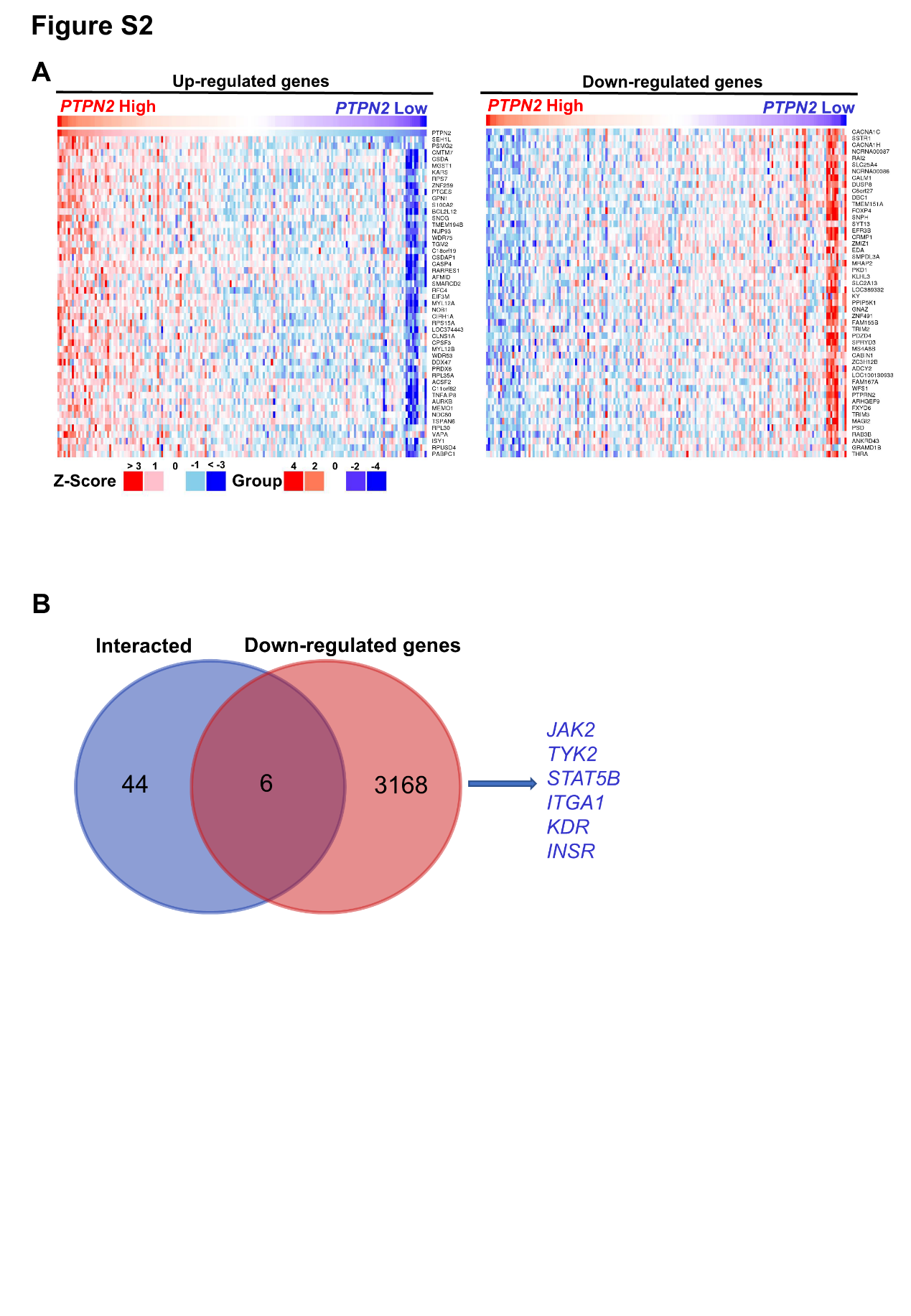


**Supplementary Figure S2. Bioinformatic analysis of *PTPN2* regulation in pancreatic cancer.** (A). The positively correlated significant genes of *PTPN2* in pancreatic cancer and the negatively correlated significant genes of *PTPN2* in pancreatic cancer. (B). online prediction the common genes in *PTPN2* PPI network and *PTPN2* negatively correlated genes.


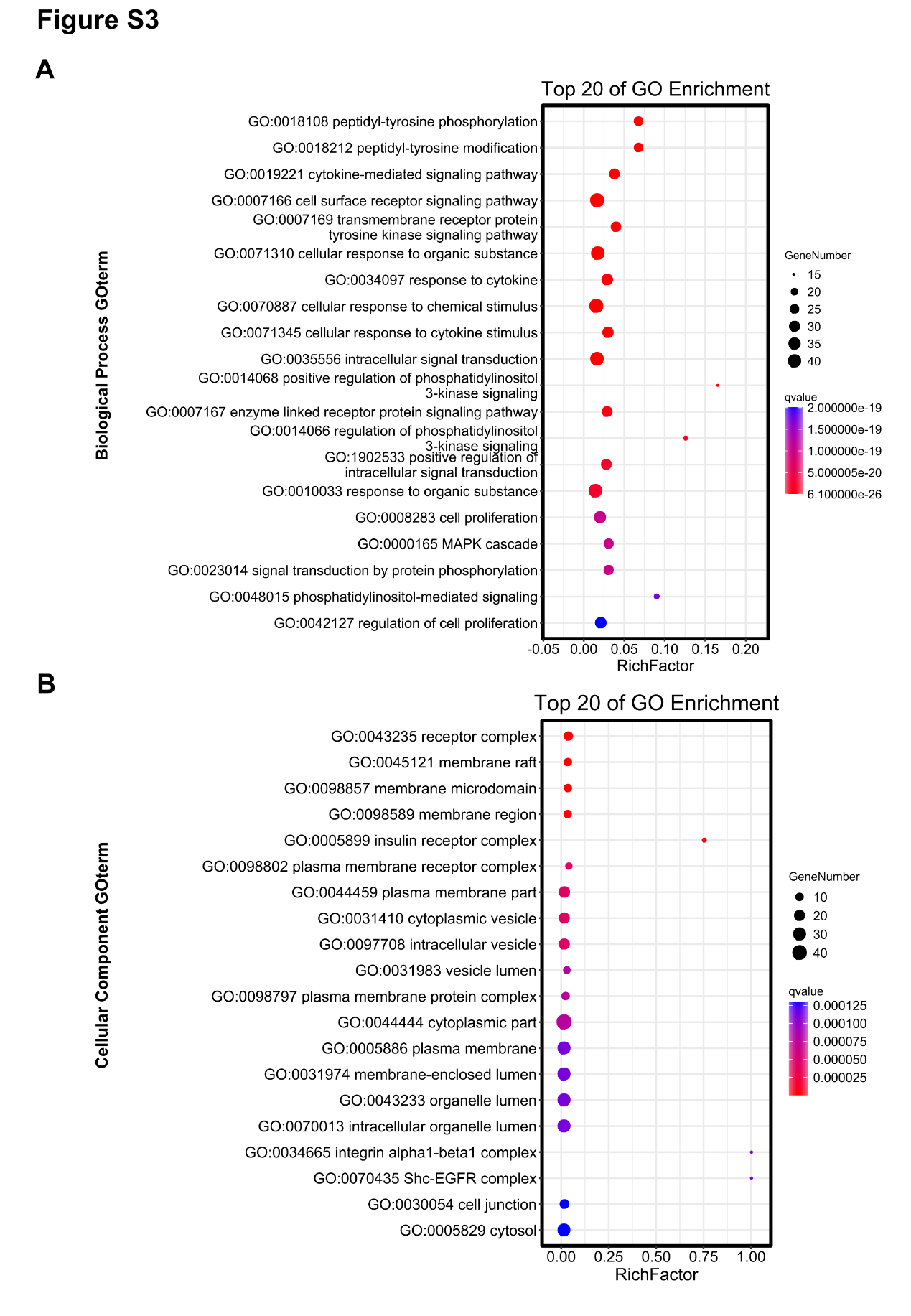


**Supplementary Figure S3. Predicting the functions of the common genes in *PTPN2* PPI network and *PTPN2* correlated genes through genetic ontology (GO) analysis.** GO enrichment analysis predicted the functional roles of the common genes in *PTPN2* PPI network and *PTPN2* correlated genes based on three aspects, including (A) biological processes, (B) cellular components, and molecular functions.

**Table S1.** Primer sequences for genes in RT-qPCR

| **Name** | **Sense (5’-3’)** | **Antisense (5’-3’)** |
| --- | --- | --- |
| *PTPN1* | TCCCTTTGACCATAGTCGGAT | GTGACCGCATGTGTTAGGCA |
| *PTPN2* | GAAGAGTTGGATACTCAGCGTC | TGCAGTTTAACACGACTGTGAT |
| *PTPN6* | GCCTGGACTGTGACATTGAC | ATGTTCCCGTACTCCGACTC |
| *PTPN9* | GATGTGCTCCGTGCCATAGA | CCTGGCAGTAAAGAGGGCAA |
| *PTPN12* | AGTTGCCTTGTTGAAGGGGAT | AGAAGGTGTCAAGATGGGTGG |
| *PTPN14* | GTGGACGAACCAAAAGCCAC | GCCCAGACAAAAGGTGCTTG |
| *PTPN23* | CCGACACTGTCAGGAACCTT | ATGTACTTGGCCCATTCTCG |
| *EGFR* | AGGCACGAGTAACAAGCTCAC | ATGAGGGACATAACCAGCCACC |
| *MET* | AACTGGTGTCCCGGATATCA | ATATTCTTTGCTCCTTGCCA |
| *JAK3*  *STAT1*  *STAT6*  *β-actin* | CTGGGCAAGGGCAACTTT  CCTGCTGCGGTTCAGTGA  TTCTGCCAAAGACCTGTCCAT  CACCATTGGCAATGAGCGGTTC | AGTCCCTCTGCTGGTCTGG  TCCACCCATGTGAATGTGATG  CTGTCCTCTACCATAGTCACA  AGGTCTTTGCGGATGTCCACGT |
